# Evaluation of liver cirrhosis and hepatocellular carcinoma using Protein-Protein Interaction Networks

**Published:** 2016-12

**Authors:** Mohammad Javad Ehsani Ardakani, Akram Safaei, Afsaneh Arefi Oskouie, Hesam Haghparast, Mehrdad Haghazali, Hamid Mohaghegh Shalmani, Hassan Peyvandi, Nosratollah Naderi, Mohammad Reza Zali

**Affiliations:** 1 Gastroenterology and Liver Diseases Research Center, Research Institute for Gastroenterology and Liver Diseases, Shahid Beheshti University of Medical Sciences, Tehran, Iran; 2 *Proteomics Research Center, Faculty of Paramedical Sciences, Shahid Beheshti University of Medical Sciences, Tehran, Iran. *; 3 *Department of Basic Sciences, Faculty of Paramedical Sciences, Shahid Beheshti University of Medical Sciences, Tehran, Iran. *; 4 *Basic and Molecular Epidemiology of Gastrointestinal Disorders Research Center, Research Institute for Gastroenterologyand Liver Diseases, Shahid Beheshti University of Medical Sciences, Tehran, Iran*; 5 *Behbood Gastroenterology and Liver Diseases Research Center, Shahid Beheshti University of Medical Sciences, Tehran, Iran*; 6 *Hearing Disorders Research Center, Shahid Beheshti University of Medical Sciences, Tehran, Iran*

**Keywords:** Cirrhosis, Hepatocellular carcinoma, Protein-Protein Interaction Network, Gene ontology

## Abstract

**Aim:**

In the current study, we analysised only the articles that investigate serum proteome profile of cirrhosis patients or HCC patients versus healthy controls.

**Background:**

Increased understanding of cancer biology has enabled identification of molecular events that lead to the discovery of numerous potential biomarkers in diseases. Protein-protein interaction networks is one of aspect that could elevate the understanding level of molecular events and protein connections that lead to the identification of genes and proteins associated with diseases.

**Methods:**

Gene expression data, including 63 gene or protein names for hepatocellular carcinoma and 29 gene or protein names for cirrhosis, were extracted from a number of previous investigations. The networks of related differentially expressed genes were explored using Cytoscape and the PPI analysis methods such as MCODE and ClueGO. Centrality and cluster screening identified hub genes, including APOE, TTR, CLU, and APOA1 in cirrhosis.

**Results:**

CLU and APOE belong to the regulation of positive regulation of neurofibrillary tangle assembly. HP and APOE involved in cellular oxidant detoxification. C4B and C4BP belong to the complement activation, classical pathway and acute inflammation response pathway. Also, it was reported TTR, TFRC, VWF, CLU, A2M, APOA1, CKAP5, ZNF648, CASP8, and HSP27 as hubs in HCC. In HCC, these include A2M that are corresponding to platelet degranulation, humoral immune response, and negative regulation of immune effector process. CLU belong to the reverse cholesterol transport, platelet degranulation and human immune response. APOA1 corresponds to the reverse cholesterol transport, platelet degranulation and humoral immune response, as well as negative regulation of immune effector process pathway.

**Conclusion:**

In conclusion, this study suggests that there is a common molecular relationship between cirrhosis and hepatocellular cancer that may help with identification of target molecules for early treatment that is essential in cancer therapy.

## Introduction

Hepatocellular carcinoma (HCC) is the one of the most common malignancy in the world (1). It usually occurs following previous liver disease, such as chronic hepatitis B or C and liver cirrhosis (LC) (2). Most HCCs develop following by chronic liver diseases (3). Multiple factors such as genetic and epigenetic changes have been reported in HCC patients (4). cDNAmicroarray studies were designed to identify abnormally expressed gene sets for HCC (5-7). On the other hand, some studies have been surveyed proteomic profile of HCC patients and have been introduced some new biomarkers (8). Many polymorphisms alter biologic pathways of carcinogenesis, including inflammation (IL1B, TNFA and TGF) (9, 10); oxidative stress (SOD2, MPO) (11, 12); DNA repair (MTHFR, XRCC3) (13, 14) and cell cycle (MDM2) (15) and TP53 (16). At least, in half of HCC patients the P53 cell cycle pathway alters with frequent TP53 mutations (12%- 48%) (17-19). Apart from genetic aberrations, alterations in protein expression have been reported such as Alpha- Fetoprotein (AFP), Des-Gamma-Carboxy (Abnormal), Prothrombin (DCP), Transforming Growth Factor-Beta (TGF-Beta), Serum Alpha-1-Fucosidase, Human Carbonyl Reductase 2, Tumor-Specific Growth Factor (TSGF), and Epidermal Growth Factor Receptor Family (EGFR) (20). At present, diagnostic methods included alpha-fetoprotein (AFP) and magnetic resonance imaging (MRI). Currently, the accepted biomarker for diagnosis of HCC is AFP; But the sensitivity and specificity of this agent are not satisfactory (21, 22). On the other way, most patients often have advanced in stage of disease at the time of diagnosis because the lack of special sign (23). Hence, there is an urgent demand to find specific biomarkers of HCC, which can be used specifically and sensitively in diagnosis as well as in prognosis and therapeutic evaluation. Liver cirrhosis (LC) and its associated complications are introduced as essential factors in morbidity and mortality worldwide (24, 25). The diagnostic methods of cirrhosis are based on the combined results of clinical and imaging examinations (24). Clinical symptoms and laboratory data of liver diseases frequently overlap; thus interpretation a differential diagnosis is difficult (26). Liver biopsy, is performed in patients with ambiguous diagnostic results. But this way is an invasive method which imposes pain to patients. For this, a non-invasive method for early diagnosis of hepatic fibrosis is needed. Some genetic liver disease that predispose to early cirrhosis with related mutations have been reported. Cystic fibrosis by altering activity of CFTR, Wilson disease by increased levels the ATP7B, hereditary hemochromatosis by iron-induced lipid peroxidation causes hepatocellular injury, Glycogen storage disease type IV by The altered stored glycogen impairs the osmotic pressure within the hepatocyte and Cholesteryl ester storage disease by Accumulation of cholesteryl are the known ones (26). Since the biological heterogeneity of liver diseases, it is difficult to distinguish HCC and other liver diseases such as cirrhosis or fatty liver, simply by the clinical symptoms or pathophysiological characteristics (27). Ideally, for timely treatment, the biomarker(s) that could recognize the fibrosis in the early stages of hepatic disorder to prevent the progression of cirrhosis to HCC is required (28). Interaction networks might give information of the functions of newly discovered proteins (29-31). The protein network analysis provides a scientific model that improves understanding of the mechanisms underlying human diseases (32-38). The centralized applications of PPI networks to disease addition to the identification of genes and proteins associated with diseases turn around on the study of network properties to find their relation to disease states. Classification of network- based disease and the identification of disease-related sub networks are another applications of the PPI network (39). Our goal is to present the PPI of cirrhosis and HCC patients versus healthy control group and comparison with each other to find common and sensitive points to introduce possible biomarker (s) in these diseases development and subsequently drug target discovering. This investigation was designed based on proteomic studies in cirrhosis and HCC patients published since 1997.

## Material and Methods

**Data Collection**

In this study, the inclusion criteria were the proteomic studies on the human species involved in the comparison between the serum or plasma of patients (cirrhosis or hepatocellular carcinoma) and healthy control. Exclusion criteria were the studies on non-human sample and the studies on samples of tissue or cell lines, saliva, CSF, and urine. It has also been eliminated the papers which were compared serum or plasma of patients with the groups except healthy control. There was no limitation in methods in proteomic study. A number of 73 papers about HCC and cirrhosis proteomic profiling were reviewed. We manually evaluated the publications and it was selected the articles in line with the above conditions. Duplicated proteins and genes were eliminated. Finely, 29 genes for cirrhosis and 63 genes for HCC were extracted. Genes and proteins were presented in tables 1 and 2. Uniprot accession number of selected genes were retrieved from web site (uniprot.org).

**Protein-Protein Interaction Analysis**

PPI network is the basic skeleton for proteins to determine their functions in the system biology (40). Specialization the interactions of proteins in a given proteome could reveal the biochemistry of the cell (41). It also helps in the identification of drug targets by introducing hubs (42). The PPI network was visualized using the Cytoscape 3.2.1 software. MINT, Reactome-FLs, databases were used for this topology visualization. We used Molecular Complex Detection (MCODE) to analyze the characteristics of the networks. The MCODE clusters was based on the topology to find the densely connected region. Gene ontology categories were analyzed to identify the function of each highly connected region that was generated by the MCODE. These include Kappa statistic ≥ 0.5, enrichment and Bonferroni step down method for probability value correction (43). The degree of functional enrichment for a given cluster was quantitatively assessed (P-value) using the ClueGO tool (44). ClueGO integrates gene ontology (GO) terms and creates a functionally organized GO/pathway term network. It can analyze genes and comprehensively visualizes functionally grouped terms (45).

**Table 1:** A number of genes in cirrhosis derived from articles include of serum proteomic profile

Protein name(references)	Uniprot code	Gene name	Protein name(references)	Uniprot code	Gene name
ApolipoproteinA-1(47)	P02647	APOA1	apolipoprotein E(46)	P02649	APOE
Haptoglobin(48)	P00738	HP	C4b-binding protein (46)alpha chain	P04003	C4BPA
Alpha1-Antitrypsin(28)	P01009	SERPINA1	Glutathione peroxidase 3(46)	P22352	GPX3
Ceruloplasmin(49)	P00450	CP	Beta-2-glycoprotein 1(28)	P02749	APOH
Transthyretin(50)	P02766	TTR	complement component 4B(46)	P0C0L5	C4B
glycoprotein 1(46, 50)	P02750	LRG1	Zinc-2-glycoprotein(28)	P25311	AZGP1
Complement factor H-related protein 1(28)	Q03591	CFHR1	Immunoglobulin gamma-2-chainc(50)	P01859	IGHG2
Apolipoprotein L1(51)	O14791	ApoL1	complement factor B(46)	P00751	CFB
Transgelin (28)	Q01995	TAGLN	complement component 4A(46)	P0C0L4	C4A
Paraoxonase/ arylesterase 1(46)	P27169	PON1	amyloid P component(46)	P02743	APCS
alpha-1-microglobulin/ bikunin (46)	P02760	AMBP	Inter-alpha-trypsin inhibitor heavy chain H4(28)	Q14624	ITIH4
haptoglobin-related protein(46)	P00739	LRG	Complement C3(28)	P01024	C3
Hemopexin(50)	P02790	HPR	1 antichymotrypsin(46) α	P01011	SERPINA3
α-1 acid glycoprotein(50)	P02763	ORM1	CD5-like antigen(50)	O43866	CD5L
			clusterin(50)	P10909	CLU

**Table 2:** A number of genes in hepatocellular carcinoma derived from articles include of serum proteomic profiling

Protein name(references)	Gene name	Uniprot code	Protein name(references)	Gene name	Uniprot code
Annexin A6(53)	ANXA6	P08133	Mannose receptor, C type 1-like 1(52)	MRC1L1	B9EJA8
Complement component 9 (53)	C9	P02748	Vascular cell adhesion protein 1(52)	VCAM1	E9PDD2
Ceruloplasmin(53)	CP	P00450	Fibrinogen β chain, isoform CRA_e(52)	FGB	D3DP13
serum amyloid A4(53)	SAA4	P35542	Proteinase 3(52)	PRTN3	D6CHE9
serum amyloid A2(53)	SAA2	P0DJI9	Hemoglobin delta-beta fusion protein(52)	HBD/HBB	Q5XTR9
Transthyretin(52, 54)	TTR	P02766	Polymeric immunoglobulin	PIGR	P01833
Clusterin(54)	CLU	P10909	receptor(52) Fos-related antigen 2(52)	FOSL2	C9JCN8
haptoglobin α2 chain(54)	HP	P00738	Cyclin-dependent kinase-like 1(52)	CDKL1	Q00532
heat-shock protein 27(55) α-fetoprotein(52)	Hsp27	P04792	cytoskeleton-associated protein5(52)	CKAP5	Q14008
Apolipoprotein A-I (46)	AFP APOA1	P02771 P02647	Transferrin receptor protein 1 (52) Transmembrane protein 200C(52)	TFRC TMEM200C	P02786 A6NKL6

**Figure 1:**
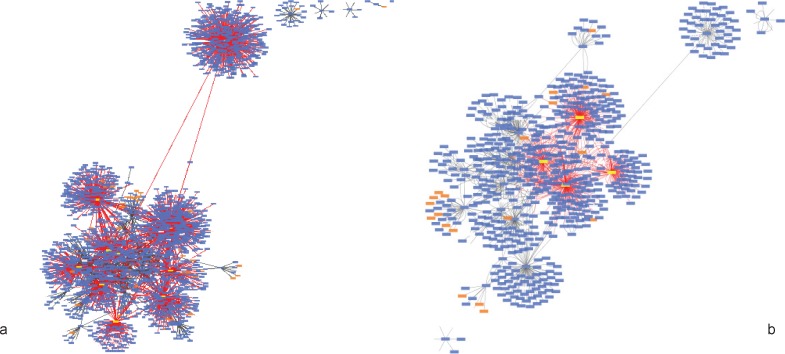
This figure shows a PPI network, which consists of 651 nodes and 1305 edges for cirrhosis (a) and 2024 nodes and 3817edges for HCC (b). The highlighted nodes APOE, TTR, CLU related to cirrhosis and TTR CLU, APOA1, TFRC, VWF, CKAP 5, A2M, ZNF648, CASP8, HSP27 for hepatocellular carcinoma

**Figure 2:**
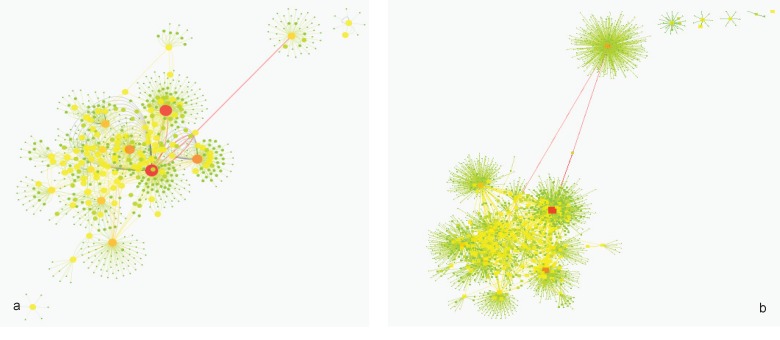
Mapping degree and betweenness parameters of candidate protein interaction network to node size and color; (a) cirrhosis patients and (b) HCC patients. More details are accessible in the text

## Results

Twenty-nine (29) genes in cirrhosis and sixty-three (63) genes in HCC with differential gene expression were distinguished via literature survey. Genes and proteins were presented in tables 1 and 2 for cirrhosis liver and HCC respectively. Hub is a node with a number of links that greatly exceeds the average (57). Cytoscape analysis revealed a great number of close interconnections that can be seen in Figure 1 (a for cirrhosis liver and b related to HCC). Based on degree values, about 15% of the initial proteins are selected as hub proteins. APOE, TTR, CLU and APOA1 as related hub proteins of cirrhosis and TTR, CLU, APOA1, TFRC, VWF, CKAP5, A2M, ZNF648, mCASP8, HSP27 as hub proteins of HCC are highlighted in figure 1 and tabulated in table 3. In figure 2 is represented based on the node size and color changes of the nodes. As the circles get bigger and their color change from green to red, their value of the degree and the betweenness centrality increase. The related subnetworks were introduced by MCODE (figure 4). Among the genes that involved in cirrhosis, APOE determined as seed while genes of HCC have no seed. ClueGo is also a Cytoscape software for gene ontology and pathway enrichment analysis. The analyzed results of MCODE by ClueGo for biological process is shown in figure 4.

**Table 3:** A number of genes with significant centrality value derived from figure 1, a for cirrhosis liver and b is related to HCC, based on two fundamental centrality properties analysis (Degree and Betweeness centrality

	**Unipr** **otcode**	**Gene** ** name**		**Degree**	**Betweenness ** **centrally**
a	P02647	APOA1		238	0.43889
	P10909	CLU		216	0.319182
	P02766	TTR		136	0.142145
	P02649	APOE		131	0.165061
b	**Uniprotcode**	**Gene name**	**Degree**		**Betweenness centrally**
	P04792	HSP27	846		0.444179
	Q14790	CASP8	544		0.162997
	Q5T619	ZNF648	400		0.361564
	Q14008	CKAP5	271		0.19395
	P02647	APOA1	238		0.159222
	P01023	A2M	230		0.131263
	P10909	CLU	216		0.101279
	P02786	TFRC	146		0.076278
	P04275	VWF	146		0.093813
	P02766	TTR	136		0.040481

**Figure 3:**
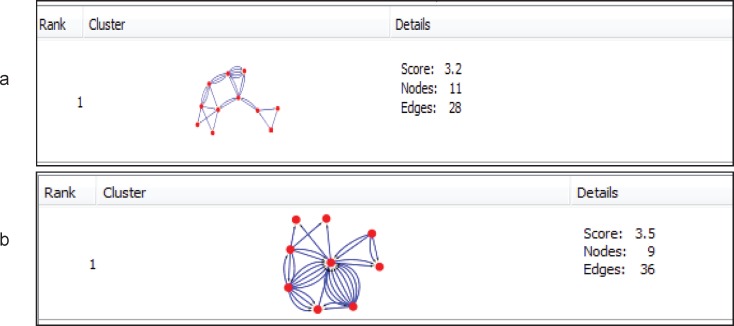
MCODE algorithm analysis demonstrates clusters based on the number of interconnections in the large network of protein-protein interactions for (a) cirrhosis disease and (b) HCC disease

## Discussion

The importance of human molecular interaction networks no summarized only in reveals protein function with their inter- relationships, even its clear view of fundamental human biology as well as disease progression, diagnosis, and treatment (58).

**Figure 4:**
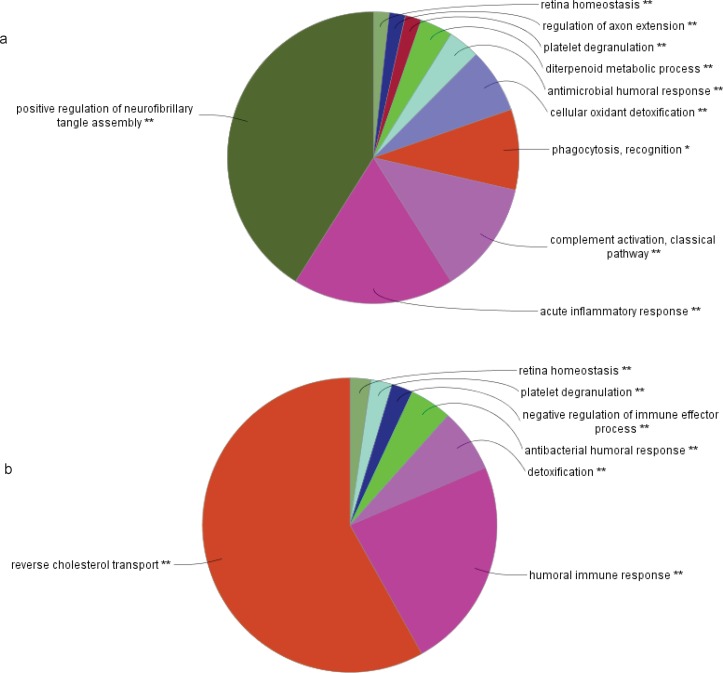
Functional distribution of biological process of modules of (a) cirrhosis and (b) HCC (b) (*P*<0.05). These include Kappa statistic ≥ 0.5, enrichment and Bonferroni step down method for probability value correction. The stars show the pathways with P-value <0.05. The pathways with two stars have more significant score rather than one star

The protein Networks analysis provides a model that elevates systems-level understanding of the mechanisms of diseases (59, 60) to analyze therapeutic drugs and their targets (61-63) and discovering the novel network-based biomarkers (64). In this study, protein network of HCC and cirrhosis patient has investigated. This analysis can lead to figure out a better understanding of the etiology of both liver disease and the specified pattern of gene expression in cirrhosis and HCC. On the other hand, therapeutic targets and diagnostic biomarkers can be accelerated by targeting the specific hub genes. Previous studies introduced cirrhosis as one of the reasons in HCC development (65) then finding molecular common points to be expected between two diseases. The importance of PPI analysis has been reported in cancer related genes (34, 36). Hub genes have virtual conception to study due to their centrality role in a PPI network (66). As represented in figure 1, protein interaction networks of HCC and cirrhosis are made up of numerous nodes that provide hub selection. APOE, TTR, CLU and APOA1 as hub protein are introduced for cirrhosis and TTR, CLU, APOA1, TFRC, VWF, CKAP5, A2M, ZNF648, CASP8 and HSP27 (HSPB1) are the related hub proteins to HCC. The all introduced hub proteins (except TTR for HCC) are bottlenecks (cut off 0.05 is used for betweenness centrality). APOA1 and CLU are the two hub-bottlenecks common between the two diseases. As it is presented in the table 3, APOA1 and CLU are characterized by the most values of degree and betweenness centralities for cirrhosis. ApoA1 is the main protein component of high density lipoprotein in plasma, which is involved in the formation of most plasma cholesterol esters (67) .This protein potently suppresses tumor growth and metastasis in multiple animal tumor models (68) . The validated changes of expression of APOA1 accompanied by a few proteins have the potential for development into high-performance tests used in the diagnosis and or monitoring of HCC and LC patients (46). CLU is a Golgi molecular chaperone involved in BAX- antiapoptotic processes, activation of the phosphatidylinositol 3-kinase/ protein kinase B pathway, promotion of angiogenesis, mediation of the nuclear factor kappa B (NF-κB) pathway and modulation of extra-cellular signal-regulated kinase (ERK) signaling. A number of biological processes, including programmed cell death (Down regulation allows for p53activation and cell death), lipid transport, membrane recycling and cell adhesion (69, 70). Serum clusterin was introduced as more specific and sensitive biomarker than AFP in distinction of HBV-cirrhosis with HCC base on HBV-cirrhosis (71). It also has been shown that clusterin may be a useful marker in the evaluation of prognosis of patients with alcoholic cirrhosis and severity of liver disease (72). CLU involved in BAX- antiapoptotic processes and it’s down regulation allows p53 activation and cell death (73). MCODE clustering algorithm (center based) demonstrates the possible presence of similar functional protein in the two PPI networks. As it is shown in figure 3 there are 2 clusters for the two PPI networks that are approximately similar. The PPI network in HCC has no seed, but in cirrhosis C4BPA is introduced as seed protein. More information about the roles of the 2 clusters in the biological processes is presented in figure 4. ClueGo provided functional annotation (BP) of the studied modules. Biological process analysis revealed some similarities between the two diseases. The significant role of the immune system and a few common pathways show the closeness of the two diseases. Some of the important proteins are involved in the terms. The highlighted roles of APOA1 in the revers cholesterol transport pathway and CLU in positive regulation of neurofibrillary tangle assembly pathway in the two major pathways of studied diseases are identified. CLU is related to reverse cholesterol transport, platelet degranulation and human immune response pathways. However, APOA1 is involved in reverse cholesterol transport, platelet degranulation and humoral immune response negative regulation of immune effector process pathways.

Protein-protein interaction analysis and pathway assessment showed a closed molecular relationship between cirrhosis and HCC. The finding pointed to the significant role of APOA1 and CLU as the common two biomarker in development of cirrhosis and HCC diseases.
